# Development and Application of Loop-Mediated Isothermal Amplification Assays for Rapid Visual Detection of *cry2Ab* and *cry3A* Genes in Genetically-Modified Crops

**DOI:** 10.3390/ijms150915109

**Published:** 2014-08-27

**Authors:** Feiwu Li, Wei Yan, Likun Long, Xing Qi, Congcong Li, Shihong Zhang

**Affiliations:** 1 College of Plant Sciences, Jilin University, Changchun 130062, China; E-Mail: lifeiwu3394@gmail.com; 2 Agro-Biotechnology Research Institute, Jilin Academy of Agricultural Sciences, Changchun 130033, China; E-Mails: jlndyanwei@126.com (W.Y.); longlikun@126.com (L.L.); licongcong1100@163.com (C.L.); 3 Plant and Soil Science Department, Texas Tech University, Lubbock, TX 79409, USA; E-Mail: qixingandpt@126.com

**Keywords:** genetically-modified organisms, loop-mediated isothermal amplification, *cry2Ab* gene, *cry3A* gene, visual detection

## Abstract

The *cry2Ab* and *cry3A* genes are two of the most important insect-resistant exogenous genes and had been widely used in genetically-modified crops. To develop more effective alternatives for the quick identification of genetically-modified organisms (GMOs) containing these genes, a rapid and visual loop-mediated isothermal amplification (LAMP) method to detect the *cry2Ab* and *cry3A* genes is described in this study. The LAMP assay can be finished within 60 min at an isothermal condition of 63 °C. The derived LAMP products can be obtained by a real-time turbidimeter via monitoring the white turbidity or directly observed by the naked eye through adding SYBR Green I dye. The specificity of the LAMP assay was determined by analyzing thirteen insect-resistant genetically-modified (GM) crop events with different *Bt* genes. Furthermore, the sensitivity of the LAMP assay was evaluated by diluting the template genomic DNA. Results showed that the limit of detection of the established LAMP assays was approximately five copies of haploid genomic DNA, about five-fold greater than that of conventional PCR assays. All of the results indicated that this established rapid and visual LAMP assay was quick, accurate and cost effective, with high specificity and sensitivity. In addition, this method does not need specific expensive instruments or facilities, which can provide a simpler and quicker approach to detecting the *cry2Ab* and *cry3A* genes in GM crops, especially for on-site, large-scale test purposes in the field.

## 1. Introduction

From the records collected from approximately 30 countries from 1996 to 2013, the global cultivated area of genetically-modified (GM) crops increased 100-fold, from 1.7 million hectares in 1996 to 175.2 million hectares in 2013, indicating that the adoption rates of GM crops were unprecedented in modern agro-technology development history [1]. Insect-resistance, deployed in GM maize, soybean, cotton, rice and poplar, has consistently been one of the most important traits in GM crops. Numerous *Bt* (*Bacillus thuringiensis*) genes, such as *cry1Ab*, *cry2Ab* and *cry3A*, encoded insecticidal crystal proteins and were the most prevalent insect-resistant genes in GM crops [2]. Right now, *cry2Ab* and *cry3A* genes are commercially used in insect-resistant GM crops. For example, the transgenic maize, MON89034, MIR604, and transgenic cotton, MON15985, developed by Monsanto, are grown widely in the world. However, since the public concern with GM food safety and environment risk, a series of legislation has been issued for genetically-modified organism (GMO) regulation and labeling in more than 50 countries and areas [3]. For example, China has implemented a mandatory labeling regulation on 17 GM products of five kinds of GM crops since 2002. Therefore, it is highly important and necessary to develop some more accurate, quicker and higher efficiency methods for GMO detection.

Currently, a variety of molecular testing methods based on exogenous nucleic acid or newly expressed protein has been established and used for GMO detection [3]. The former includes polymerase chain reaction (PCR), real-time PCR, DNA fingerprinting and microarray [4,5,6,7], and the latter includes enzyme-linked immunosorbent assay (ELISA), lateral flow strip, liquid chromatography and western blot analysis [2,8,9,10]. Among all of the methods above, conventional and quantitative real-time PCR methods based on nucleic acid sequences are the most widely used methodologies for identifying or quantifying GM crops and their derivate products, because of the high specificity, sensitivity and reproducibility [11,12]. However, the PCR assay requires high-precision instruments and trained personnel for its implementation, which may partially limit its application for *in situ* testing [13].

Loop-mediated isothermal amplification (LAMP) is an outstanding nucleic acids amplification procedure developed by Notomi *et al.* in 2000 [14]. The LAMP assay employs four specially-designed primers (two inner primers and two outer primers), which can recognize six independent regions on the target DNA, showing extremely high specificity. The LAMP reaction can be carried out at a constant temperature between 60 and 65 °C by one type of enzyme with strand displacement activity within 60 min [15]. Thus, a simple isothermal instrument, such as a water bath, is adequate for LAMP amplification. Furthermore, the LAMP result can be easily monitored by using a turbidimeter or determined visually with the naked eye by adding a fluorescent dye, such as SYBR Green I, into the reaction mixture [16,17]. Compared to conventional PCR, the LAMP technique is easy and quick with higher specificity, sensitivity and effectivity. Thus, this new method has been widely used in clinical diagnosis [18,19,20,21], food safety inspection [22,23] and species identification [24,25]. Due to its competitive advantage, the LAMP method was used increasingly for GMO detection [26,27,28,29,30,31,32,33,34]. However, this method has never been used for *cry2Ab* and *cry3A* detection. The goal of this study is to employ the LAMP method on *cry2Ab* and *cry3A* gene detection in GM crops, which is simpler and quicker, especially for on-site, large-scale testing purposes in the field.

## 2. Results and Discussion

### 2.1. Extraction of Genomic DNA from Samples

Genomic DNA was successfully extracted from all samples by using the Plant Genome DNA Extraction Kit (Tiangen Biotech Co., Ltd., Beijing, China). The DNA purity and quantity were confirmed by using the ND1000 Spectrophotometer. To confirm if the extracted DNA were suitable for LAMP and PCR reaction, three endogenous reference genes, the *zSSIIb* gene for maize, the *ACP* gene for cotton and the *SPS* gene for rice, were selected as the internal controls and detected according to [35,36,37]. The observation of the prospective amplification fragments of the endogenous reference genes in all samples indicated that the extracted genomic DNA purity and quantity were adequate for LAMP and PCR analysis (Supplementary Figure S1).

### 2.2. Design of the Loop-Mediated Isothermal Amplification (LAMP) Primers for cry2Ab and cry3A Genes

**Figure 1:**
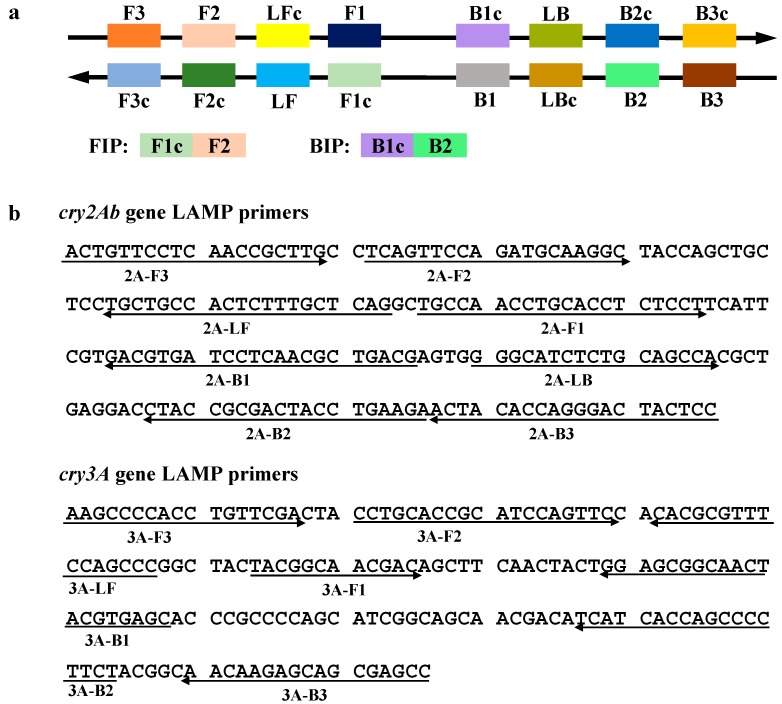
Primer design for loop-mediated isothermal amplification (LAMP) assays. (**a**) The schematic diagram showing the location of each primer; and (**b**) Partial coding sequences of the *cry2Ab* and *cry3A* genes used to design the primers. LAMP primers are indicated by arrows.

The LAMP method relies on auto-cycling strand displacement DNA synthesis, which is carried out by a DNA polymerase with high strand displacement activity, such as *Bst* DNA polymerase, and a set of four specially-designed primers that recognize a total of six distinct regions on each of the target DNA sequences [14]. Nagamine *et al.* [38] demonstrated that the LAMP reaction could be accelerated by using loop primers. In this study, one or two additional loop primers were also used. The full lengths of *cry2Ab* and *cry3A* genes re 1902 bp (GenBank Accession No. AR260579) and 1803 bp (GenBank Accession No. AX712169), respectively. In this study, the LAMP primers of the *cry2Ab* gene, including two outer primers (2A-F3 and 2A-B3), two inner primers (2A-FIP and 2A-BIP) and two loop primers (2A-LF and 2A-LB), were designed according to the 196-bp-specific sequence of the *cry2Ab* gene from GM maize MON89034. The *cry3A* gene LAMP primers, including two outer primers (3A-F3 and 3A-B3), two inner primers (3A-FIP and 3A-BIP) and one loop primer (3A-LF), were targeted to a 176-bp-specific sequence of the *cry3A* gene from GM maize MIR604. Forward inner primers (FIP) contained the F1c (complementary to F1) and the F2 sequences. Backward inner primers (BIP) contained the B1c (complementary to B1) and the B2 sequences. The primers used in this study are listed in Table 1, and the schematic diagram of the LAMP primer design and detailed locations of LAMP primers in the target DNA sequences are shown in Figure 1.

**Table 1:** Oligonucleotide primers used in this study.

Primers Name	Sequence (5'–3')	Target	Amplicon Size (bp)	Reference
2A-F3	ACTGTTCCTCAACCGCTTG	*cry2Ab* gene	196	This work
2A-B3	GGAGTAGTCCCTGGTGTAGT			
2A-FIP	AGGAGAGGTGCAGGTTGGCACTCAGTTCCAGATGCAAGGC			
2A-BIP	GACGTGATCCTCAACGCTGACGTCTTCAGGTAGTCGCGGTAG			
2A-LF	CTGAGCAAAGAGTGGCAGCA			
2A-LB	GGGCATCTCTGCAGCCA			
3A-F3	AAGCCCCACCTGTTCGA	*cry3A* gene	176	This work
3A-B3	GGCTCGCTGCTCTTGTTG			
3A-FIP	AGTTGAAGCTGTCGTTGCCGTACCTGCACCGCATCCAGTTC			
3A-BIP	GGAGCGGCAACTACGTGAGCAGAAGGGGCTGGTGATGA			
3A-LF	GGGCTGGAAACGCGTGT			

### 2.3. Specificity of the LAMP Assays

To evaluate the specificity of this developed LAMP assay for the *cry2Ab* and *cry3A* genes, thirteen GM events and one non-GM mixture sample (including non-GM maize, cotton and rice) were collected and used. The selected GM events in this study contained ten of the most important insect-resistant genes (*cry1Ab*, *cry1Ac*, *cry1F*, *cry1Ab/Ac*, *cry1A.105*, *cry2Ab*, *cry3A*, *cry3Bb*, *cry34Ab1* and *cry35Ab1*), which have had a commercialized application for a long time and which represented the foremost insect-resistant transgenic crops all over the world. The prospective LAMP and conventional PCR results of these GM crops are shown in Table 2. One hundred nanograms of corresponding template DNA were used in each LAMP and conventional PCR reaction mixture.

**Table 2:** The prospective results of LAMP and conventional PCR detecting the *cry2Ab* and *cry3A* genes in GM crops with different *Bt* genes ^α^.

Species	GM Crops	Contained *Bt* Genes	*cry2Ab*		*cry3A*	
			LAMP	PCR	LAMP	PCR
Maize	Bt11	*cry1Ab*	−	−	−	−
	Bt176	*cry1Ab*	−	−	−	−
	MON810	*cry1Ab*	−	−	−	−
	TC1507	*cry1F*	−	−	−	−
	MON89034	*cry1A.105*, *cry2Ab*	+	+	−	−
	MON863	*cry3Bb*	−	−	−	−
	MON88017	*cry3Bb*	−	−	−	−
	MIR604	*cry3A*	−	−	+	+
	59122	*cry34Ab1*, *cry35Ab1*	−	−	−	−
Cotton	MON531	*cry1Ac*	−	−	−	−
	MON15985	*cry1Ac*, *cry2Ab*	+	+	−	−
Rice	TT51-1	*cry1Ab/Ac*	−	−	−	−
	KF-6	*cry1Ab*	−	−	−	−

^α^ “**+**” indicates a positive result; “−” shows a negative result.

**Figure 2:**
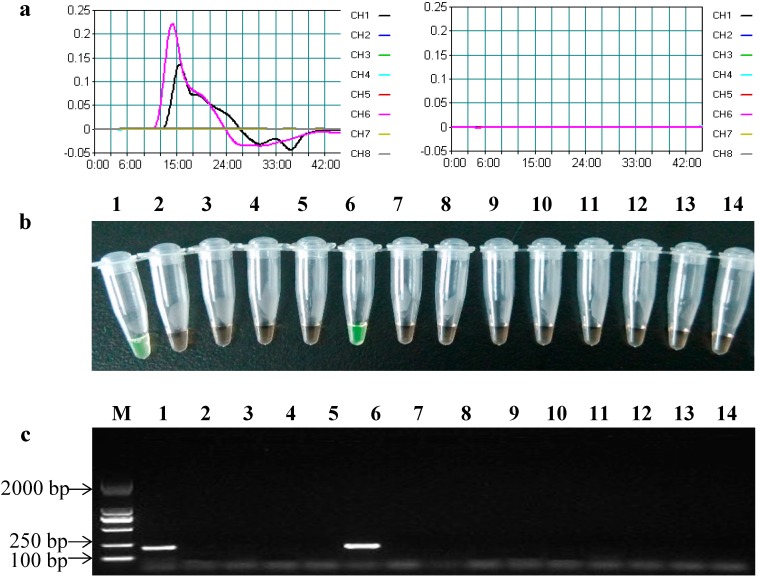
Specificity of the LAMP assay and conventional PCR for detecting the *cry2Ab* gene. (**a**) The time threshold curves of the LAMP assay observed by using the LA-320C turbidimeter. (**Right**) CH1, GM maize MON89034; CH2, GM maize MON810; CH3, GM maize Bt11; CH4, GM maize Bt176; CH5, GM cotton MON531; CH6, GM cotton MON15985; CH7, GM rice KF-6; CH8, GM rice TT51-1; (**Left**) CH1, GM maize MON863; CH2, GM maize MON88017; CH3, GM maize MIR604; CH4, GM maize TC1507; CH5, GM maize 59122; CH6, non-GM crop mixture; (**b**) The LAMP assay results using SYBR Green I dye; and (**c**) The conventional PCR results. Lane M, DL2000 DNA marker (TaKaRa Biotechnology Co., Ltd., Dalian, China); Lanes 1–14, MON89034, MON810, Bt11, Bt176, MON531, MON15985, KF-6, TT51-1, MON863, MON88017, MIR604, TC1507, 59122, non-GM crop mixture.

A Loopamp Realtime turbidimeter (LA-320C) was employed to detect the LAMP products in the *cry2Ab* and *cry3A* gene LAMP assays. The typical time threshold curve indicating turbidity values was obtained in the reaction mixtures using MON89034 maize and MON15985 cotton genomic DNA samples as templates, indicating that these reactions were LAMP-positive reactions, while no typical time threshold curve was observed in other GM and non-GM crops, indicating that these reactions were LAMP-negative reactions (Figure 2a). In addition, when adding SYBR Green I dye into the LAMP reaction mixture, a positive color of green was observed only in the LAMP reaction containing MON89034 maize or MON15985 cotton genomic DNA (Figure 2b). In fact, only MON89034 maize and MON15985 cotton contained the *cry2Ab* gene in all of these samples. Thus, this established LAMP assay had excellent specificity for the *cry2Ab* gene.

Conventional PCR was normally used in the qualitative detection of GM crops, due to the low cost, high specificity and simple procedures in previous studies [3]. As a comparison, all of the above samples were tested by conventional PCR using the outer primers (F3 and B3). A consistent result was obtained between the conventional PCR method and the LAMP assay (Figure 2c). In addition, in order to ensure the accuracy of PCR amplification products, the *cry2Ab* gene amplicon from MON89034 maize was purified and sequenced (Sangon Biotech Co., Ltd., Shanghai, China). The sequencing results revealed that this 196-bp amplicon was 100% consistent with the expected sequence (the data are not shown).

**Figure 3:**
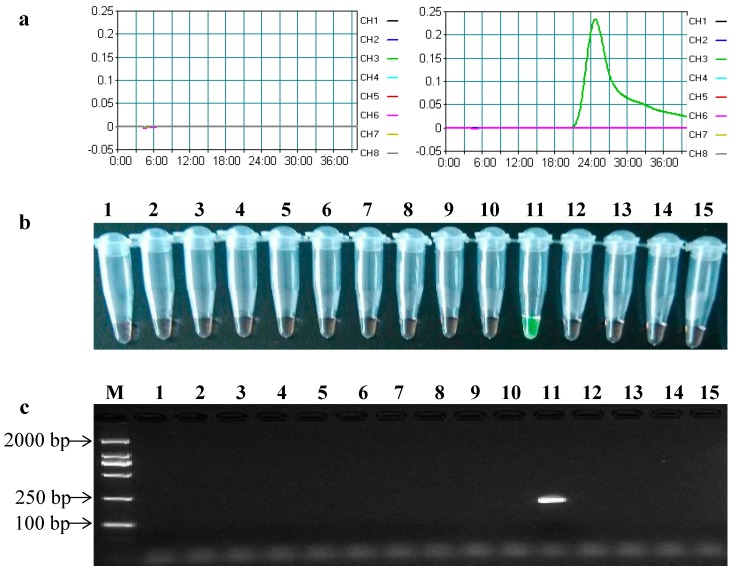
Specificity of the LAMP assay and conventional PCR of the *cry3A* gene. (**a**) The time threshold curves of the LAMP assay observed by using the LA-320C turbidimeter. (**Right**) CH1, GM maize MON89034; CH2, GM maize MON810; CH3, GM maize Bt11; CH4, GM maize Bt176; CH5, GM cotton MON531; CH6, GM cotton MON15985; CH7, GM rice KF-6; CH8, GM rice TT51-1; (**Left**) CH1, GM maize MON863; CH2, GM maize MON88017; CH3, GM maize MIR604; CH4, GM maize TC1507; CH5, GM maize 59122; CH6, non-GM crop mixture; CH7, no-template control (NTC); (**b**) The LAMP assay results using SYBR Green I dye; and (**c**) The conventional PCR results. Lane M, DL2000 DNA marker; Lanes 1–15, MON89034, MON810, Bt11, Bt176, MON531, MON15985, KF-6, TT51-1, MON863, MON88017, MIR604, TC1507, 59122, non-GM crop mixture, NTC.

Similarly, in the LAMP and conventional PCR assays for detecting the *cry3A* gene, positive results were obtained only in the reaction mixture using MIR604 maize genomic DNA as a template (Figure 3). Actually, MIR604 maize was the only GM crop containing the *cry3A* gene among all of the samples above. These data demonstrated that this developed LAMP assay had a high specificity for reliably detecting the target transgenes.

### 2.4. Sensitivity of the LAMP Assays

**Figure 4:**
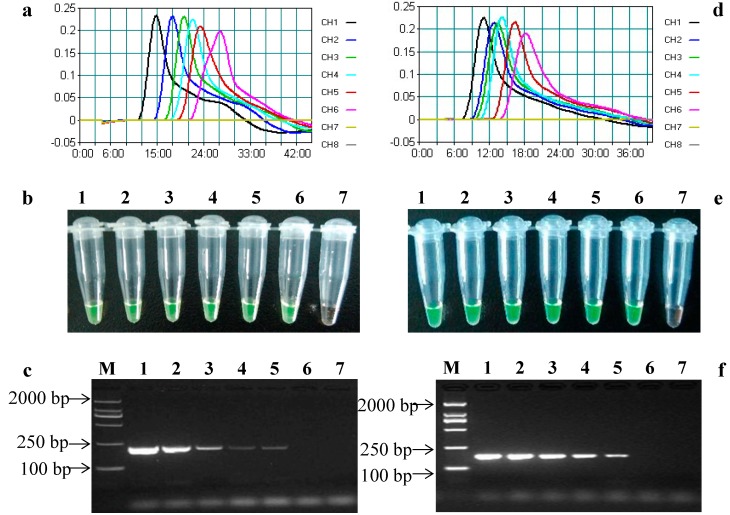
Sensitivity of LAMP assays and conventional PCR for detecting the *cry2Ab* and *cry3A* genes. (**a**) The time threshold curves of *cry2Ab* LAMP assay observed by using the LA-320C turbidimeter. CH1-6, MON89034 genomic DNA samples with final concentrations of 10^4^, 10^3^, 10^2^, 10, 5 and 1 copy/μL; CH7, NTC; (**b**) The *cry2Ab* LAMP assay results using SYBR Green I dye; (**c**) Conventional PCR results of the *cry2Ab* gene. Lane M, DL2000 DNA marker; Lanes 1–6, MON89034 genomic DNA samples with final concentrations of 10^4^, 10^3^, 10^2^, 10, 5 and 1 copy/μL; Lane 7, NTC; (**d**) The time threshold curve of *cry3A* LAMP assays observed by using the LA-320C turbidimeter. CH1-6, MIR604 genomic DNA samples with final concentrations of 10^4^, 10^3^, 10^2^, 10, 5 and 1 copy/μL; CH7, NTC; (**e**) The *cry3A* LAMP assay results using SYBR Green I dye; and (**f**) Conventional PCR results for the *cry3A* gene. Lane M, DL2000 DNA marker; Lanes 1–6, MIR604 genomic DNA samples with final concentrations of 10^4^, 10^3^, 10^2^, 10, 5 and 1 copy/μL; Lane 7, NTC.

The limit of detection (LOD) is the minimum concentration or amount of the analyte in a sample that can be detected reliably [32]. To test the LOD of the established LAMP assays, genomic DNA extracted from 100% GM maize MON89034 and MIR604 were serially diluted with 0.1× TE buffer to final concentrations of 10^4^, 10^3^, 10^2^, 10, 5 and 1 copy/μL, according to the haploid genome size of maize [39]. Five microliter-diluted DNA samples were used as templates in each reaction. In the *cry2Ab* LAMP assay, the typical time threshold curve and color change from orange to green were observed in all of the dilutions (Figure 4a,b), indicating that the absolute LOD of *cry2Ab* LAMP assay was about as low as five copies of haploid maize genomic DNA. Meanwhile, the same LODs were obtained in the *cry3A* LAMP assay (Figure 4d,e).

As a comparison, conventional PCR was performed using the outer primers (F3 and B3) with the same diluted genomic DNA sample, and the LOD of conventional PCR for both the *cry2Ab* and *cry3A* genes was approximately 25 copies (Figure 4c,f). These results indicated that this established LAMP assay was more sensitive than the conventional PCR method, which was similar to the previous studies [26,27]. According to the original report of the LAMP assay, a few copies of DNA could be rapidly amplified to 10^9^ in less than one hour [14]. The high sensitivity of LAMP could sometimes lead to false-positive amplification, due to cross-contamination, especially during agarose gel electrophoresis. Therefore, it would be more practical and feasible to perform the LAMP assay by closed-tube endpoint detection instead of gel electrophoresis.

## 3. Experimental Section

### 3.1. Plant Material

Seed samples of thirteen insect-resistant GM crop events were used in this study. GM maize events MON89034, MON810, MON863 and MON88017 and GM cotton events MON531 and MON15985 were developed and kindly provided by Monsanto Company (St. Louis, MO, USA). GM maize events MIR604, Bt11 and Bt176 were developed and kindly provided by Syngenta Company (Basel, Switzerland). GM maize events TC1507 and 59122 were developed and kindly provided by Dow Agro Sciences LLC (Indianapolis, IN, USA) and Pioneer Hi-Bred International (Johnston, IA, USA). GM rice event TT51-1 was developed and kindly provided by Huazhong Agricultural University (Wuhan, China). GM rice event KF-6 was developed and kindly provided by the4 Institute of Genetics and Developmental Biology, Chinese Academy of Sciences (Beijing, China). Non-GM maize, soybean, cotton and rice were purchased from a local supermarket (Changchun, China).

### 3.2. DNA Isolation

Genomic DNA was isolated from seed powder using the Plant Genome DNA Extraction Kit (Tiangen Biotech Co., Ltd., Beijing, China) according to the manufacturer’s instructions and then quantified using the ND1000 Spectrophotometer (NanoDrop Technologies Inc., Wilmington, DE, USA). The genomic DNA was separated on 1% (*w*/*v*) agarose gel in 1× TAE stained with GenFinder dye (Biov Biotechnology Co., Xiamen, China). The final genomic DNA concentration was adjusted to 20 ng/μL by using 1× TE solution.

### 3.3. Primers Design

The *cry2Ab* gene-specific LAMP primers were designed based on the exogenous nucleotide sequence of GM maize line MON89034 [40], and the *cry3A* gene-specific LAMP primers were designed based on the exogenous nucleotide sequence of GM maize event MIR604 [41]. All of the LAMP primers were designed by using an online software Primer Explorer V4 [42] and synthesized by Sangon Biotech Co., Ltd. (Shanghai, China). The scheme of the LAMP primers designed for target sequences are shown in Figure 1. All primers used in this work are described in Table 1.

### 3.4. LAMP Assay

The LAMP assay was carried out with the Loopamp^®^ DNA Amplification kit (Eiken China Co., Ltd, Shanghai, China). The 25-μL volume reaction mixture contained 1× Reaction Mix (20 mM Tris–HCl (pH 8.8), 10 mM KCl, 10 mM (NH_4_)_2_SO_4_, 8 mM MgSO_4_, 0.1% Tween 20, 0.8 M betaine, 1.4 mM each dNTPs), 0.2 μM each outer primer (F3 and B3), 1.6 μM each inner primer (FIP and BIP), 0.8 μM each loop primer (LF and LB), 8 U Bst DNA polymerase (Eiken China Co., Ltd, Shanghai, China) and 5 μL template DNA. Each reaction was initially incubated at 63 °C for 60 min using a turbidimeter or a thermostatic water bath, followed by heating at 80 °C for 5 min to inactivate the polymerase and terminate the reaction. LAMP products were kept at 4 °C until analyzed. The LAMP assay was performed in triplicate for each sample, and ddH_2_O was used instead of the template DNA in the no template control (NTC).

### 3.5. Analysis of LAMP Products

The white turbidity caused by the existence of magnesium pyrophosphate, the LAMP amplification by-product of each reaction mixture, was simultaneously and continuously monitored by using a Loopamp Realtime turbidimeter, LA-320C (Eiken Chemical Co., Ltd., Tochigi, Japan). The LAMP-positive reaction mixture became turbid, while the negative reaction without amplified products still remained clear. In addition, 1 μL 1000× SYBR Green I dye (Invitrogen, Ltd., Carlsbad, CA, USA) was dropped at the center of the reaction tube cover before incubation and briefly centrifuged into LAMP products for visualization purposes by the naked eye. The samples that turned to a green color were regarded as positive, while those remaining orange were considered negative.

### 3.6. Conventional PCR Assay

Conventional PCR was also carried out to compare the specificity and sensitivity with the established LAMP methods. The outer primers (F3 and B3) of each LAMP assay were employed for the conventional PCR. The 25-μL volume reaction mixture contained 1× PCR buffer (10 mM Tris–HCl (pH 8.3), 50 mM KCl, 1.5 mM MgCl_2_), 0.2 mM each dNTP, 0.2 μM each primer (F3 and B3), 1 U Taq DNA polymerase (TaKaRa Biotechnology Co., Ltd., Dalian, China) and 5 μL template DNA. Each reaction was initially denatured at 95 °C for 5 min; followed by 35 cycles of 94 °C for 30 s, 56 °C for 30 s and 72 °C for 30 s; and a final extension at 72 °C for 5 min using a C1000 Thermal cycler (Bio-Rad Laboratories, Hercules, CA, USA). PCR products were separated on 2% (*w*/*v*) agarose gel in 1× TAE stained with GenFinder dye (Biov Biotechnology Co., Xiamen, China) and photographed using a GelDoc XR+ Imager (Bio-Rad Laboratories, Hercules, CA, USA).

## 4. Conclusions

In this study, a visual and rapid LAMP assay was successfully developed for the detection of the *cry2Ab* and *cry3A* genes in GM crops. The results from turbidity monitoring and SYBR Green I staining indicated that the LAMP assays are highly specific for the target genes. Compared to the conventional PCR method, which requires at least 2–3 h of PCR reaction and product detection time, the LAMP method established in this study could be finished within one hour. In addition, the LAMP reaction results could be determined by the naked eye, whereas conventional PCR requires time-consuming electrophoretic analysis.

Furthermore, the limits of detection of established LAMP assays for identifying the *cry2Ab* and *cry3A* genes were as low as five copies, much less than the conventional PCR method (25 copies), indicating that the LAMP assay had a higher sensitivity than the conventional PCR approach.

In conclusion, this established LAMP assay is quick, accurate and cost effective, with high specificity and sensitivity. In addition, this method does not need specific expensive instruments or facilities, which can provide a simpler and quicker approach to detecting the *cry2Ab* and *cry3A* genes in GM crops, especially for on-site, large-scale testing purposes in the field.

## Supplementary Files

Supplementary File 1
