# Supplementary material for: Development and Application of Loop-Mediated Isothermal Amplification Assays for Rapid Visual Detection of cry2Ab and cry3A Genes in Genetically-Modified Crops
Source: Int J Mol Sci. 2014 Aug 27;15(9):15109–21. doi: 10.3390/ijms150915109 (PMC4200818; doi:10.3390/ijms150915109)

Supplementary Information

Figure S1. The conventional PCR results for endogenous reference genes. (**a**) Maize endogenous reference gene *zSSIIb*; (**b**) cotton endogenous reference gene *ACP*; and (**c**) rice endogenous reference gene *SPS*. Lane M, DL2000 DNA marker (TaKaRa Biotechnology Co., Ltd., Dalian, China); Lanes 1–15, Bt11, Bt176, MON810, TC1507, MON89034, MON863, MON88017, MIR604, 59122, MON531, MON15985, TT51-1, KF-6, non-GM crop mixture with maize, cotton and rice, blank control.

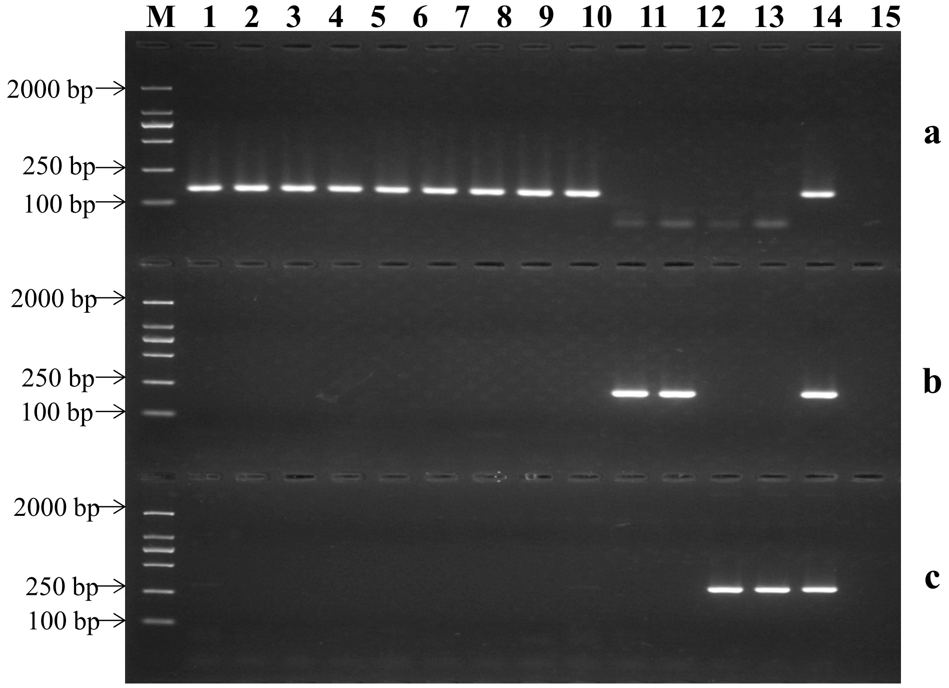

Supplement: Supplementary File 1 [file ijms-15-15109-s001.pdf]
